# Biosurveillance of avian influenza and Newcastle disease viruses in the Barda region of Azerbaijan using real time RT-PCR and hemagglutination inhibition

**DOI:** 10.3389/fmicb.2015.01128

**Published:** 2015-11-06

**Authors:** Shalala Zeynalova, Fizuli Guliyev, Mahira Vatani, Bahruz Abbasov

**Affiliations:** ^1^Virology Department, Republican Veterinary LaboratoryBaku, Azerbaijan; ^2^Virology Department, Barda Zonal Veterinary LaboratoryBaku, Azerbaijan

**Keywords:** avian influenza, Newcastle disease, Azerbaijan, environmental surveillance, live bird market

## Abstract

The Azerbaijan State Veterinary Control Service (SVCS) has conducted active serological surveillance for avian influenza (AI) in poultry since 2006, when the first outbreak of AI H5N1 occurred in Azerbaijan. Samples are collected from September to May annually and tested using a hemagglutination inhibition (HI) assay to detect antibodies against H5 AI viruses. HI testing is also performed for Newcastle disease virus (NDV) upon request, but since this method cannot distinguish between natural infections and immune responses to vaccination, all positive results require follow-up epidemiological investigations. Furthermore, blood collection for the surveillance program is time-intensive and can be stressful to birds. In order to improve the national surveillance program, alternative sampling and testing methodologies were applied among a population of birds in the Barda region and compared with results of the national surveillance program. Tracheal and cloacal swabs were collected instead of blood. Rather than testing individual samples, RNA was pooled to conserve resources and time, and pools were tested by real-time reverse transcription polymerase chain reaction (rRT-PCR). Environmental sampling at a live bird market was also introduced as another surveillance mechanism. A total of 1,030 swabs were collected, comprising tracheal, and cloacal samples from 441 birds and 148 environmental surface samples from farms or the live bird market. During the same time, 3,890 blood samples were collected nationally for the surveillance program; 400 of these samples originated in the Barda region. Birds sampled for rRT-PCR were likely different than those tested as part of national surveillance. All swab samples tested negative by rRT-PCR for both AI and NDV. All blood samples tested negative for H5 by HI, while 6.2% of all samples and 5% of the Barda samples tested positive for exposure to NDV. Follow-up investigations found that positive samples were from birds vaccinated in the previous month. This study demonstrated that taking swabs was quicker and less invasive than blood collection. Results of rRT-PCR testing were similar to HI testing for H5 but also ruled out infection with all influenza type A viruses and not just H5. In addition, rRT-PCR testing was able to rule out active infections with NDV.

## Introduction

Influenza A virus subtype H5N1, also known as highly pathogenic avian influenza (HPAI) or simply H5N1, is a virus that infects birds and can cause serious illness and death in humans (Liu et al., 2005; Alexander and Capua, 2009; Belser et al., 2011). The first case of human H5N1 was recorded in 1997 during a poultry outbreak in Hong Kong. After its re-emergence in 2003 and 2004, the virus spread from Asia to Europe and Africa, where it has since resulted in over 800 human infections and 400 deaths (World Health Organization [WHO], 2005; Gilsdorf et al., 2006; Belser et al., 2011). Migratory birds can spread H5N1 and other avian viruses over long distances (OIE World Organization for Animal Health, 2012). AI outbreaks in poultry have a negative impact on livelihoods, local economies, and international trade in affected countries. The circulation of H5N1 in poultry also threatens public health, since this strain has the potential to cause serious disease in people and is capable of mutating into a form that is more transmissible among humans, increasing the risk of a pandemic and mass mortality event.

Influenza virus subtypes other than H5N1 that circulate in poultry and other animals also pose a threat to public health. For example, influenza A viruses of the H7 hemagglutinin type can be highly pathogenic in birds and must be reported to the OIE World Organization for Animal Health (2012) when they are detected. H7 AI viruses are also known to infect humans. A novel H7N9 virus emerged in 2013 that has caused a total of 571 laboratory-confirmed infections and 212 deaths to date as of February 23, 2015 (World Health Organization [WHO], 2015). H9 viruses also can cause infections in birds and people (Lin et al., 2000; Iqbal et al., 2009; Poovorawan et al., 2013). An H9N2 viruse identified in poultry has been found to be responsible for contributing six genes to the H7N9 influenza virus that emerged in 2013 and is known to be pathogenic in people (Pu et al., 2014).

Newcastle disease (ND) is a highly contagious viral avian disease that is a differential diagnosis for HPAI as it causes clinical symptoms similar to those of HPAI, including damage to the respiratory, intestinal, and nervous systems of infected birds. ND has been classified by the OIE as an especially dangerous disease (Group A) on account of its ability to adversely affect the health of large populations of birds. Newcastle disease virus (NDV), the causative agent of ND, is known to infect over 200 species of birds (World Organization for Animal Health, 2008). The severity of disease varies by host species and strain of virus; the fatality rate in birds infected with NDV has reached 100% in past outbreaks (Agayeva and Zeynalova, 2011). In recent years, the reported incidence of ND has increased worldwide; outbreaks have been detected in Europe, America, Asia, and Africa (Gilsdorf et al., 2006).

Azerbaijan has recorded outbreaks of both viruses in poultry, most recently in 2006. In February 2006, H5N1 AI was first identified in Azerbaijan among wild birds along the coast of the Caspian Sea near Baku. Later that month, infections were detected in poultry on farms in the northeastern and southern regions of the country. The first cases of human H5N1 infection were recorded in March, and by December 2006 the disease had spread throughout Azerbaijan, resulting in eight confirmed human cases, five of which were fatal. The outbreaks were ultimately traced to wild birds and domestic poultry in the Gilazi and Bilasuvar rayons (Agayeva and Zeynalova, 2011; Belser et al., 2011). Two outbreaks of NDV were also recorded in 2006 (Agayeva and Zeynalova, 2011).

Following the 2006 outbreak of H5N1, the Azerbaijan State Veterinary Control Service (SVCS) initiated active serological monitoring for AI in poultry. Azerbaijan has a total of approximately 17 million poultry, of which about 70% are privately owned and 30% are commercially owned. The majority of poultry are considered village or backyard poultry as categorized by the FAO definition of poultry production systems (Food and Agriculture Organization [FAO], 2004). Under the SVCS program, blood samples from poultry are tested for the presence of antibodies against H5 by the hemagglutination inhibition (HI) assay using OIE-standardized reagents. Twelve Zonal Veterinary Laboratories (ZVLs) and Regional Veterinary Offices in Azerbaijan are engaged in this surveillance program. Sample collection from domestic birds occurs between September and May each year and testing is performed at the Republican Veterinary Laboratory (RVL). When the Regional Veterinary Offices submit samples for H5 testing, they may also request ND disease testing, which is performed by HI using OIE-standardized reagents. ND testing is requested for approximately half of the samples submitted.

The study described herein was undertaken to investigate sampling and testing methodologies that might be better alternatives to those currently deployed for AI and ND serosurveillance in Azerbaijan. HI testing of HPAI is subtype-specific, but the current surveillance system only tests for H5 viruses (State Veterinary Control Service, 2013), and therefore will fail to detect immune responses to other subtypes (including known human-pathogenic strains such as H7 and H9). In addition, HI testing requires blood samples, which are time-intensive and more invasive to collect than other types of biological samples (such as tracheal and cloacal swabs).

The specific goals of this study were to use rRT-PCR to test tracheal and cloacal swab samples for AI and ND viral RNA; to screen samples for the influenza A matrix gene so as to detect all subtypes known to be pathogenic to humans; to implement sample pooling as a way to reduce the costs of testing; and to introduce environmental sampling in live bird markets as a way to increase the chances of detecting disease in an area where species of birds from many locations are commingled. The OIE Terrestrial Manual states that while virus culture is the “gold standard” diagnostic test for identifying virus, culture is laborious and time insensitive. Real-time RT-PCR is the diagnostic method of choice in many laboratories for identifying the presence of antigen (OIE World Organization for Animal Health, 2012). Several studies have shown the rRT-PCR has been shown to have the added benefit of being able to pool samples as a way to save money on reagents for both AI and ND testing (Fereidouni et al., 2012; Spackman et al., 2013). Live bird markets have long been known to be a source of influenza and NDVs. Influenza A H5, H7, and H9 viruses, which can affect both poultry and people, have all been identified in poultry or the environment of live bird markets (Liu et al., 2003; Ge et al., 2009; Wan et al., 2011; Lee et al., 2013; Waziri et al., 2014). NDV has also been identified in live bird markets (Jibril et al., 2014; Mulisa et al., 2014). Environmental sampling of live bird markets has been shown to be an effective way to assess influenza contamination (Indriani et al., 2010). In one study, PCR of environmental samples was a more effective method for identifying the presence of influenza virus contamination than viral culture (Horm et al., 2013).

In order to investigate sampling and testing methodologies that might be better alternatives or supplements to those methods currently deployed for AI and ND serosurveillance in Azerbaijan, domestic poultry were sampled between September 2013 and April 2014 using new methodologies aimed at detecting the presence of antigen. The results of testing were compared with results of the ongoing national surveillance aimed at detecting immune response against AI A H5 viruses.

## Materials and Methods

### Site Selection

Project activities were centered in Barda, a region with a documented history of H5N1 and ND cases in birds (Agayeva and Zeynalova, 2011). The region is regularly exposed to migratory birds because it is situated between the Mingechevir water reservoir to the north and the Goygol and Agh Gol National Parks to the west and the southeast, respectively. According to the Azerbaijan Ministry of Ecology and Natural Resources, these areas comprise part of the migratory flyway for several wild bird species. Moreover, Barda city hosts the largest live animal market in Azerbaijan. Finally, of all the rayons participating in the SVCS surveillance program for AI, Barda historically generates the most samples and routinely requests the most ND testing.

Project investigators conducted an initial sample collection trip in May 2013, followed by eight more collection trips between September 2013 and April 2014. Samples were collected at the live bird market in Barda city during each of the nine collection trips. In addition, samples were collected from farms or households in 17 of 110 villages and Barda city. To randomly select villages for sampling, every village in the Barda rayon was assigned a number, and numbers were randomly selected from this list using the RAND function in Microsoft Excel. To identify farms for sampling, investigators would randomly choose one of four directions from the center of each selected village by spinning a bottle from the center and choosing the direction that the bottle pointed in. Every other farm or household was selected in the chosen direction, up to a total of three farms or households with poultry. If fewer than three farms or households existed in a given direction within the boundaries of a village, another direction was chosen randomly to continue the study. The geo-coordinates (latitude and longitude) of the farms/households and market where sampling occurred were captured using an eTrex10 GPS unit (Garmin, Olathe, KS, USA) and recorded in an Excel spreadsheet.

### Sample Collection

**FIGURE 1:**
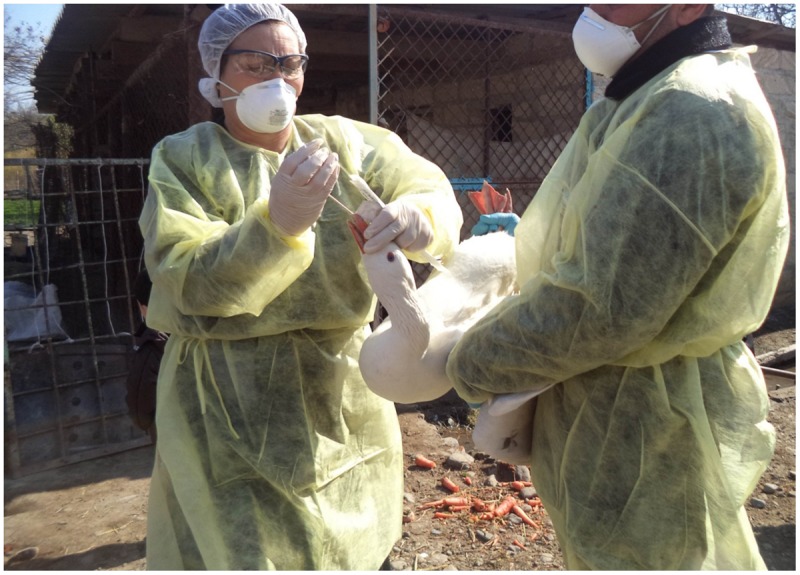
**Collecting swab samples from birds at Kelenterli village**.

Samples for testing by rRT-PCR were collected from domestic birds (ducks, geese, turkeys, and chickens) found at the selected farms/households from Sector 4 production systems (village or backyard poultry with minimal biosecurity and birds consumed locally) as categorized by the Food and Agriculture Organization [FAO] (2004). At the live bird market, the collection teams solicited the voluntary participation of poultry vendors during six site visits. Cloacal and tracheal samples were collected from each of the birds enrolled in this study (**Figure 1**). Cloacal sampling was performed by gently inserting a sterile cotton-tipped swab (Puritan, Inc., Guilford, ME, USA) approximately 1 cm into the vent. The tip of the swab was rolled along the interior surface of the cloaca before it was removed and placed into a sterile conical tube for transport to the laboratory. To collect tracheal samples, a sterile cotton-tipped swab was inserted as deeply as practicable into the oropharynx of the bird being tested. The investigators made a special effort to bring the sterile swab in contact with the trachea and not simply the back of the oral cavity. The swab was withdrawn and placed into a sterile conical tube without media for transport to the laboratory. During the collection procedures, the birds were restrained manually without sedation, anesthesia, or other medications. Sampling activities were conducted according to SVCS guidelines which are the same as the (Food and Agriculture Organization [FAO], 2006) guidelines and no birds were injured during collection.

Environmental swabs were collected in areas where birds were held while awaiting sale at the live bird market. A dry, sterile, cotton-tipped swab without media was used to swab cages, tables, and wooden boards contaminated with the excretions of caged birds. Each sample, regardless of its source, was placed into an individual sterile conical tube to avoid any cross-contamination that could confound the results of the diagnostic testing. All samples were transported to the Barda ZVL on ice on the day of collection, where they were stored at –20°C pending transport to the RVL in Baku for testing at the end of the sampling period.

Sample size was calculated to estimate the prevalence of AI in poultry in the Barda region considering all poultry as one population due to the minimal biosecurity in place for backyard farms and the live poultry market. As the actual prevalence of AI or ND in this population was not known, the investigators assumed a prevalence of 50% to conservatively determine the appropriate sample size. From this assumption, the minimum number of birds to sample was estimated to be 385 (with a 95% confidence interval and 5% allowable error). Sample size was calculated using WinEpiscope 2.0 (Thrusfield et al., 2001). All birds available at a selected farm were swabbed, which on average was approximately four birds per farm.

This project was conducted with approval by the Azerbaijan State Veterinary Control Service (SVCS) Scientific Committee, which reviewed the project proposal for scientific and ethical concerns, including the use of animals. This work was funded through a grant from the Cooperative Biological Engagement Program (CBEP); the Medical Research and Materiel Command (MRMC) Animal Care and Use Review Office (ACURO) determined that the protocols described surveillance methods, and therefore Institutional Animal Care and Use Committee (IACUC) oversight was unnecessary. Additionally, bird sampling procedures were deemed to cause less stress to the birds than those normally conducted as part of the national surveillance for H5 AI viruses. No birds were harmed during any part of the sampling associated with this project. All sample collection activities were completed in accordance with appropriate biosafety procedures. Participants donned appropriate personal protective equipment for the collection process, including disposable gowns, gloves, hair nets, goggles, N95 respirators, and shoe covers.

### Sample Management and Preparation

Each sample was systematically assigned a unique identification number at the time of collection to facilitate matching of tracheal and cloacal swabs collected from the same bird. A record sheet was created to track the samples. The information captured on the specimen records included the sample identification number, species sampled (turkey, ducks, geese, chickens), and sample origin (i.e., cloaca, trachea, or environmental surface). If the bird had any outward clinical signs of illness such as discharge or diarrhea, this was also noted. The samples were shipped on ice to the RVL at the end of each monthly collection trip; total transit time between Barda and Baku is approximately 4 h. Upon arrival at the RVL, the samples were stored at –20°C pending RNA extraction, which typically occurred within 1 week of sample arrival. Each sample was extracted individually and then pooled for viral RNA testing with rRT-PCR.

### Real-time RT-PCR Analysis

All 1,030 samples collected for rRT-PCR were extracted using the QIAamp Viral RNA Mini Kit (Qiagen, Inc., Gaithersburg, MD, USA) according to the manufacturer’s instructions. After the RNA was extracted from individual samples, a portion of the viral RNA from up to five samples of the same epidemiological unit and sample type was pooled. A total of 213 pools were created from the samples collected over the course of the project.

Real-time RT-PCR amplification was performed using the SuperScript II One-Step RT-PCR System (Invitrogen Inc., Carlsbad, CA, USA); primer/probe sets (Sigma–Aldrich Inc., Germany); and a Lightcycler 2.0 instrument (Roche Diagnostics, Indianapolis, IN, USA). Invitrogen Platinum Taq DNA polymerase kits (Life Technologies, Carlsbad, CA, USA) were used to prepare the master mix for each pathogen (influenza A virus or NDV). Real-time RT-PCR testing for a conserved region of the influenza A matrix gene was used to screen samples generically for all influenza A virus (AIV) strains (Spackman et al., 2002). Real-time RT-PCR testing for the Fusion gene was conducted to detect NDV as previously described (Wise et al., 2004; Alexander and Capua, 2009). The specific forward and reverse primers used for testing both AIV and NDV are shown in **Table 1**. A total of 4 μL of extracted pooled RNA was used in each 20 μL reaction. Positive and negative controls were included in each run and each sample was run in duplicate. Had any samples tested positive for the AIV matrix gene, H5, H7, and/or H9 subtype-specific primers and probes would have been used to characterize the virus subtype.

**Table 1:** Primer/probe sequences and target pathogens and genomes.

Primer/Probe	Sequence^∗^	Pathogen target	Genome target
**Influenza A virus**			
AI gene M forward primer	5′ AGATGAGTCTTCTAACCGAGGTCG 3′	AIV	Matrix
AI gene M reverse primer	5′ TGCAAAAACATCTTCAAGTCTCTG 3′		
Probe AI gene M	FAM-TCAGGCCCCCTCAAAGCCGA –TAMRA		
**Newcastle disease virus**			
Forward primer	5′ GGTGAGTCTATCCGGARGATACAAG 3′	NDV	Fusion
Reverse primer	5′ AGCTGTTGCAACCCCAAG 3′		
Probe	5′ [FAM]AAGCGTTTCTGTCTCCTTCCTCCA [BHQ] 3′		

*^∗^Fluorescent dye abbreviations: 6FAM, 6-Carboxyfluorescein; TAMRA, tetramethylrhodamine; BHQ1 = Black Hole Quencher^®^-1.*

### National Active Surveillance and Vaccination Program for AI H5 and ND

Since 2006, the Azerbaijan SVCS has conducted an annual serosurveillance program in which all 12 regional ZVLs participate (**Table 2**). Each ZVL is responsible for collecting samples from 4 to 5 rayons to ensure that surveillance occurs throughout the country. Field veterinarians collect serum samples from domestic fowl, and tissue samples are collected from dead wild birds in Azerbaijan’s national parks and nature reserves. Veterinarians submit collected samples to the local ZVL, which in turn sends them to RVL for testing. Testing for AI H5 (and ND, when requested) is conducted by the SVCS according to standard HI protocols (Alexander and Capua, 2009; OIE World Organization for Animal Health, 2012).

**Table 2:** Total number of samples tested for avian influenza and Newcastle disease viruses during active surveillance from 2011 to 2014.

Year	Domestic poultry blood tested for H5	Wild bird internal organs tested for H5	Domestic poultry blood tested for NDV
2011	8,752	110	4,020
2012	6,192	79	3,000
2013	7,353	18	3,700
2014	7,074	127	4,000

The SVCS conducts an annual vaccination program against ND using two live lentogenic virus vaccines, including H and LaSota vaccines. No vaccination is conducted for AI.

## Results

### Sample Collection

**FIGURE 2:**
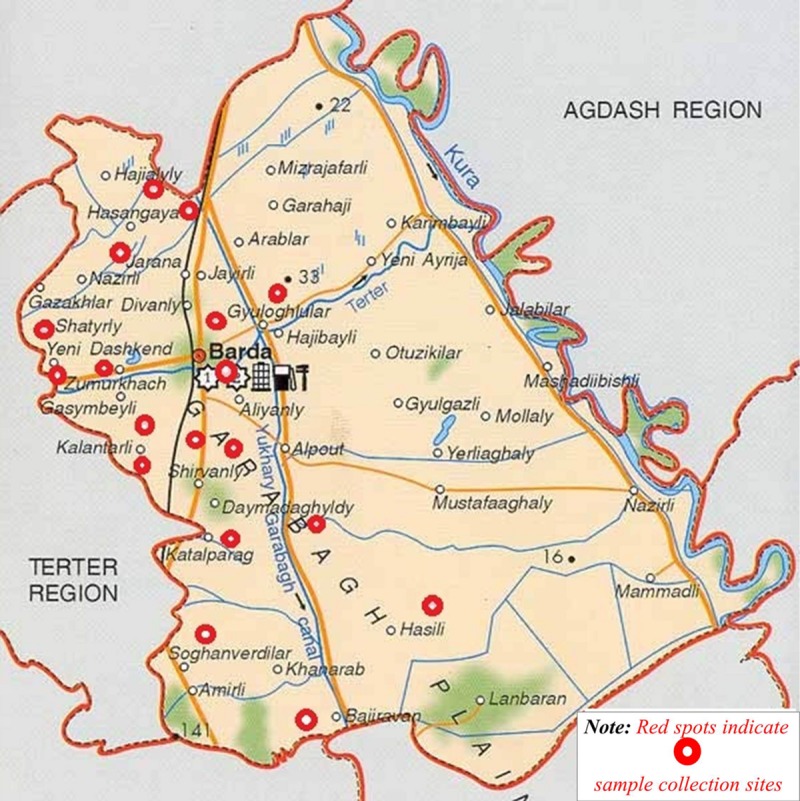
**Red circles indicate sampling sites in the Barda region of Azerbaijan**.

Overall, 1,030 swab samples were collected from birds and the environment for testing by rRT-PCR over nine sampling events (one event each in May, September, October, November, and December 2013, and January and April 2014; two events in March 2014). A total of 441 birds were sampled including chickens (*n* = 341), turkeys (*n* = 54), geese (*n* = 24), and ducks (*n* = 22). Of the 441 birds sampled, 89 were from the live market while 352 were from private farms in the 17 villages and the city of Barda (**Figure 2**). Of 441 birds sampled, 301 (68%) of these birds had been previously vaccinated against ND with either the LaSota or the H vaccine. One bird exhibited nasal discharge at the time of sampling; all other birds were clinically healthy. The investigators also collected 134 environmental swabs from the live bird market and 14 environmental samples from farms (**Table 3**).

**Table 3:** Number and type of samples collected from the Barda region (2013–2014).

Collection period	Location	# of farms	# of samples collected				
			Trachea	Cloaca	Environ	Samples per location	Total
23–24 May 2013	Live bird market	N/A	15	15	0	30	104
	Households in central Barda	N/A	15	15	2	32	
	Zumurkhan village	5	20	20	2	42	
26–27 September 2013	Live bird market	N/A	10	10	0	20	108
	Imamgulubeyli village	5	15	15	4	34	
	Chemenli village	8	26	26	2	54	
23–25 October 2013	Live bird market	N/A	18	18	7	43	163
	Qara-Yusifli village	10	40	40	0	80	
	Bala-Qecher village	10	20	20	0	40	
13–15 November 2013	Live bird market	N/A	10	10	9	29	153
	Qahramanli village	9	30	30	2	62	
	Shatirli village	10	30	30	2	62	
18–20 December 2013	Live bird market	N/A	16	16	8	40	100
	Garana village	3	15	15	0	30	
	Guloglular village	5	15	15	0	30	
27–29 January 2014	Live bird market	N/A	20	20	8	48	90
	Seyif Yusifli village	2	10	10	0	20	
	Hadjali village	3	11	11	0	22	
14 March 2014	Live bird market	N/A	0	0	30	30	30
16–19 March 2014	Ketelparaq village	3	18	18	0	36	252
	Soganverdiler village	3	18	18	0	36	
	Muganli village	3	18	18	0	36	
	Kelenterli village	3	17	17	0	34	
	Qasimbeyli village	3	17	17	0	34	
	Alakadirli village	3	17	17	0	34	
	Live bird market	N/A	0	0	42	42	
19 April 2014	Live bird market	N/A	0	0	30	30	30
**Total**		88	441	441	148	1,030	1,030

### Real-time RT-PCR Analysis

A total of 213 RNA pools were tested for NDV and for the influenza A virus matrix gene by rRT-PCR. None of the pooled samples were positive for either of the agents (**Table 4**). No specific subtype testing for H5, H7, or H9 was necessary.

**Table 4:** rRT-PCR screening results of pooled samples by collection month.

Sampling month and year	Number of Pools	Newcastle disease results	Influenza A matrix gene results
May 2013	34	Negative	Negative
September 2013	22	Negative	Negative
October 2013	33	Negative	Negative
November 2013	29	Negative	Negative
December 2013	20	Negative	Negative
January 2014	20	Negative	Negative
March 2014	50	Negative	Negative
April 2014	5	Negative	Negative
**Total**	213		

### National Active Surveillance and Vaccination Program for AI H5 and ND

Between September 2013 and May 2014, the national surveillance program collected a total of 3,890 samples for AI virus testing throughout Azerbaijan; 400 of these originated from domestic birds in the Barda region, but were likely different than those collected for rRT-PCR testing. None of the 3,890 samples were positive for H5 AIV by the HI assay. A total of 240 samples (6.2%) were positive for NDV overall; 20 (5%) of the 400 samples from Barda were positive. According to 2014 SVCS data, 19,631,361 domestic birds were inoculated against NDV with the H vaccine and 18,639,580 birds were vaccinated with the LaSota vaccine.

## Discussion

The results from the rRT-PCR testing conducted in this study aligned with the results of the SVCS HI testing program for H5, which has not detected any birds infected with AI H5 since 2006. The rRT-PCR test used in this study targeted the influenza matrix A gene. Because this gene is common to all influenza A viruses, it can be used to screen for several different strains of influenza type A viruses. A positive result on the rRT-PCR assay for the matrix gene would be an indication for additional testing to identify the specific influenza strain present in the sample. The AI HI assay only detects AI H5 and does not test for the presence of other hemagglutinin types. Although the results appear to be consistent, a direct comparison between the results was not possible because the samples collected in the national surveillance program and the Barda study were not from the same birds. Regardless, the failure of two different testing strategies to detect H5 suggests that this strain of AIV is not present in the Barda region.

This study showed that real-time rRT-PCR testing could be a valuable addition to the national surveillance program. Among the samples from Barda that SVCS tested for NDV, 5% had detectable antibodies. The detection of antibodies indicates that the birds were exposed to NDV antigen either through natural infection or exposure to a vaccine that triggered an immune response detectable by serology testing. The SVCS vaccinates birds annually with live lentogenic virus vaccines, and a positive serology test result would not be unexpected. No large-scale die-offs or symptoms consistent with NDV were reported in Azerbaijan during the surveillance period. Coupled with the lack of positive rRT-PCR test results, this indicates that none of the birds tested were actively infected. In the absence of clinical evidence of a ND outbreak, it is likely that birds were exposed to NDV antigen through vaccination rather than natural infection. Further epizootological investigation conducted by the district veterinarian concluded that the positive results detected were likely the result of the vaccine administered a month before sampling. Additional studies comparing the results of rRT-PCR and serological assays on samples collected from the same birds would help to establish rRT-PCR testing as a viable addition to conventional serology testing to rule out active infections, or as an alternative testing method to minimize false positive test results.

Other studies have similarly applied both HI and rRT-PCR to surveillance systems, using HI to detect seropositivity and using rRT-PCR to detect active infections. A surveillance program for H5 and H7 AIV in Poland used RT-PCR to investigate active infections following a positive HI result (Pikula et al., 2014). Out of 45,000 serum samples, nine geese or ducks were positive for H5 or H7 on HI. When tested by RT-PCR, all were negative. The conclusion of the authors was that a low pathogenic AIV had previously infected some birds, but that there was no actively circulating viral infections (Pikula et al., 2014). Another surveillance program aimed at evaluating AIVs in waterfowl in Spain identified 1.1 and 0.3% of birds seropositive for H5 and H7. Of 47 samples that tested positive by both HI and ELISA, all tested negative by rRT-PCR (Jurado-Tarifa et al., 2014). A study using both HI and RT-PCR to investigate ND in caged birds in Tehran, found that RT-PCR was more sensitive for detection of active carriers than was the HI test when 35 of 335 tested positive by RT-PCR but were not positive by HI (Madadgar et al., 2013).

There are several potential explanations for the lack of any positive rRT-PCR results. First, there may be no AIV or NDV circulating in the study area; this possibility is supported by the absence of clinical evidence of disease caused by either virus. Most birds in this study appeared healthy; only one farm had one sick bird which tested negative for both AIV and NDV. Alternatively, flaws in the study design and execution may have interfered with detection of positive samples, despite efforts to maximize the success of the project. For example, the villages selected for the study may not have been conducive to disease detection due to previous vaccination efforts against NDV. Excluding vaccinated birds from studies is problematic because the vaccination status of individual birds may be difficult to ascertain. In a real-world setting, such as the live bird market or a village farm, the owner of a given bird may not be fully aware of its provenance, especially when live birds are bought and sold in an uncontrolled market. In the absence of a controlled study, future investigations could consider two alternative research directions. One approach would be to collect samples in isolated regions where no vaccinated birds are known to exist. Alternatively, samples for rRT-PCR testing and for serology testing could be collected from the same bird. Birds that are positive by serology tests but negative by rRT-PCR are likely to have been vaccinated; birds that are positive by both assays are most likely infected, either because they were not vaccinated or because the vaccine was ineffective.

This project introduced environmental testing as a new method to detect viral diseases that may be present at live bird markets. Such environments can present an opportunity for continued viral transmission as healthy birds come into contact with birds that may be infected. Environmental testing is not invasive, so it is more likely to be accepted by vendors concerned about the adverse effects that sampling could have on their birds. Environmental sampling is also safer for veterinary health officers, since it avoids the need to directly handle and sample live birds. Although the utility of environmental testing could not be verified in this study due to the lack of positive results, this approach has been validated as a method for virus detection in similar studies (Indriani et al., 2010).

This study demonstrated that swabs are a safe and easy way to collect samples from live birds, and that environmental sampling at live bird markets merits further consideration for inclusion into the Azerbaijani surveillance program. Real-time RT-PCR testing should also be considered as an addition or substitution to the program, because it can be used to identify all influenza type A viruses (including H7 and H9 viruses known to be pathogenic to humans) and can help to rule out active infection of NDV in birds testing falsely positive as a result of vaccination.

## Conflict of Interest Statement

The authors declare that the research was conducted in the absence of any commercial or financial relationships that could be construed as a potential conflict of interest.
